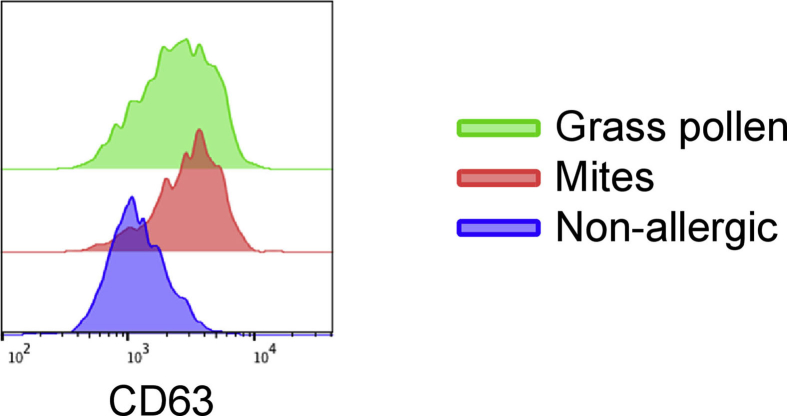# Increased expression levels of CD300c on basophils from allergic individuals^☆^

**DOI:** 10.1016/j.waojou.2019.100060

**Published:** 2019-09-30

**Authors:** Joana Vitallé, Iñigo Terrén, Ane Orrantia, Aritza Segurola, Yolanda Seras, Pedro M. Gamboa, Francisco Borrego, Olatz Zenarruzabeitia

**Affiliations:** a Immunopathology Group, Biocruces Bizkaia Health Research Institute, Barakaldo, Bizkaia, Spain; b Allergology Service, Cruces University Hospital, Barakaldo, Bizkaia, Spain; c Ikerbasque, Basque Foundation for Science, Bilbao, Bizkaia, Spain

**Keywords:** CD300c, CD300a, Basophils, Dust mites allergy, Grass pollen allergy

*To the Editor*:

Immunoglobulin E (IgE)-mediated allergy refers to the adverse reaction in some patients caused by the crosslinking of the high-affinity IgE receptor (FcεRI) on basophils and mast cells by allergen-specific IgEs. Once the contact with allergens is established, immunomodulatory therapeutic agents to prevent the onset of allergic symptoms are currently quite limited. Blocking the interaction of specific IgE with the FcεRI that is present in basophils and mast cells is an important target in anti-allergic therapy. Clinical responses have been observed after the administration of omalizumab, a humanized anti-IgE antibody, to patients with food allergy, which correlated with the suppression of degranulation of basophils.^1^, ^2^ These results indicate that actions aimed to blocking or modulating the IgE/FcεRI axis represent a promising strategy for the treatment of allergy and anaphylaxis. Therefore, in order to develop new immunomodulatory therapies, it is very important to characterize cell surface receptors capable of modulating IgE-mediated activation threshold in basophils and mast cells.^3^

The human CD300 family of receptors consists of 8 receptors expressed in both myeloid and lymphoid lineages.^4^ It has been recently demonstrated that the phosphatidylserine and phosphatidylethanolamine binding CD300c receptor acts as a co-stimulatory molecule during basophil activation through the IgE/FcεRI axis. CD300c cross-linking significantly augmented IgE-mediated basophils degranulation and cytokine production in a process involving increased calcium mobilization and phosphorylation of signaling intermediates such as protein tyrosine kinase (Syk) and extracellular signal–regulated kinases (ERK). Moreover, it was observed that basal expression levels of CD300c on basophils from IgE-dependent cow's milk allergic children are higher than those from healthy control children, suggesting that CD300c could be used as a biomarker in the diagnosis of the IgE-dependent allergic pathology.^5^

In order to further assess the clinical relevance of those findings, we have studied the expression of CD300c on basophils from patients with two IgE-dependent allergies. Clinical features from patients are shown in Table 1. We collected peripheral blood samples from 3 different cohorts: 1) adults with dust mites allergy (n = 36), 2) adults with grass pollen allergy (n = 22), and 3) non-allergic control individuals (n = 26).

**Table 1:** Clinical data of allergic individuals

Patient	Gender	Age (years)	Symptoms	Severity	Total IgE (kU/L)	IgE Der p (kU/L)	IgE Phl pe (kU/L)
HCAC_001	Female	60	ASTHMA + RHINITIS	Mild	3526	>100	–
HCAC_002	Male	19	ASTHMA + RHINITIS	Mild	215	27.6	–
HCAC_003	Male	34	ASTHMA + RHINITIS	Severe	168	18.4	–
HCAC_004	Female	15	ASTHMA + RHINITIS	Moderate	1012	>100	–
HCAC_005	Female	43	RHINITIS	Mild	133	39.8	–
HCAC_006	Male	20	RHINITIS	Mild	526	>100	–
HCAC_007	Female	31	ASTHMA + RHINITIS	Mild	254	39.2	–
HCAC_008	Male	28	ASTHMA + RHINITIS	Mild	618	>100	–
HCAC_009	Female	36	RHINITIS	Mild	175	18.5	–
HCAC_010	Female	20	ASTHMA + RHINITIS	Moderate	362	81.8	–
HCAC_011	Female	31	ASTHMA + RHINITIS	Mild	52	nd	–
HCAC_012	Male	41	ASTHMA + RHINITIS	Severe	168	4.46	–
HCAC_013	Female	40	ASTHMA + RHINITIS	Mild	1247	>100	–
HCAC_014	Female	36	ASTHMA + RHINITIS	Severe	416	59.2	–
HCAC_015	Female	78	ASTHMA + RHINITIS	Mild	384	17.3	–
HCAC_016	Female	26	ASTHMA + RHINITIS	Mild	445	49.6	–
HCAC_017	Female	78	ASTHMA + RHINITIS	Mild	361	5.47	–
HCAC_018	Female	46	RHINITIS	Moderate	199	11.6	–
HCAC_019	Female	15	RHINITIS	Mild	ND	80.1	–
HCAC_020	Female	25	ASTHMA + RHINITIS	Mild	666	nd	–
HCAC_021	Female	41	ASTHMA + RHINITIS	Mild	260	40.8	–
HCAC_022	Male	18	ASTHMA + RHINITIS	Mild	213	27.3	–
HCAC_023	Female	32	ASTHMA + RHINITIS	Mild	458	34.8	–
HCAC_024	Female	20	RHINITIS	Mild	37	10.6	–
HCAC_025	Female	49	ASTHMA + RHINITIS	Mild	18	4.46	–
HCAC_026	Female	23	RHINITIS	Mild	38	1.76	–
HCAC_027	Male	27	RHINITIS	Mild	44	13.4	–
HCAC_028	Female	31	RHINITIS	Mild	67	8.02	–
HCACGR_001	Male	20	RHINITIS	Mild	782	56.7	–
HCACGR_002	Female	15	ASTHMA + RHINITIS	Mild	449	69.2	–
HCACGR_003	Male	17	RHINITIS	Mild	721	44.4	–
HCACGR_004	Female	16	ASTHMA + RHINITIS	Mild	2882	>100	–
HCACGR_006	Male	21	ASTHMA + RHINITIS	Moderate	1553	>100	–
HCACGR_007	Female	30	ASTHMA + RHINITIS	Mild	691	35.8	–
HCACGR_008	Female	47	RHINITIS	Mild	481	7.2	–
HCACGR_009	Female	29	RHINITIS	Mild	302	nd	12.6
HCACGR_010	Female	45	ASTHMA + RHINITIS	Mild	301	39.8	–
HCGR_001	Female	36	RHINITIS	Mild	493	–	73.2
HCGR_002	Male	44	RHINITIS	Mild	14	–	1.23
HCGR_003	Male	28	ASTHMA + RHINITIS	Mild	324	–	51.8
HCGR_004	Female	34	RHINITIS	Mild	1237	–	15.9
HCGR_005	Male	28	RHINITIS	Mild	924	–	28.4
HCGR_006	Female	37	RHINITIS	Mild	Nd	–	Nd
HCGR_007	Male	31	RHINITIS	Mild	17,3	–	5.35
HCGR_008	Male	43	RHINITIS	Mild	148	–	2.54
HCGR_009	Female	30	ASTHMA + RHINITIS	Mild	228	–	12.5
HCGR_010	Female	56	ASTHMA + RHINITIS	Mild	11,01	–	2.1
HCGR_011	Female	11	RHINITIS	Mild	140	–	26.8
HCGR_012	Female	24	ASTHMA + RHINITIS	Mild	988	–	89.6
HCGR_013	Male	46	RHINITIS	Mild	242	–	29
HCGR_014	Female	36	RHINITIS	Mild	374	–	23.6
HCGR_015	Male	48	RHINITIS	Mild	107	–	21.2
HCGR_016	Female	54	RHINITIS	Mild	44,6	–	7.32
HCGR_017	Male	44	RHINITIS	Mild	34,7	–	6.04
HCGR_018	Male	25	RHINITIS	Mild	136	–	16.3
HCGR_020	Male	57	RHINITIS	Mild	409	–	57.3
HCGR_021	Female	16	RHINITIS	Mild	436	–	>100
HCGR_022	Male	33	RHINITIS	Mild	194	–	72.2

Dr p: *Dermatophagoides pteronyssinus*; Phl p: *Phleum pratense*

To identify basophils among the peripheral blood mononuclear cells (PBMCs), we have used a staining strategy based on the expression of the surface receptor CD123 and the absence of human leukocyte antigen - DR isotype (HLA-DR) (shown in the article's online supplementary Figure S1). First, we analyzed the expression of CD63, a basophil activation marker which is rapidly mobilized to the cell surface by polyclonal anti-IgE and allergens, as well as other degranulation stimuli.^6^ In agreement with previous publications,^7^ we observed that subjects with dust mites and/or grass pollen allergy show a significant increase in the median fluorescence intensity (MFI) of CD63 compared with basophils from non-allergic subjects, indicating that they are activated *in vivo* (Fig. 1A and online supplementary Figure S2). We cannot confirm that the increased expression on CD63 is exclusively due to a higher expression on basophils, and that other possibilities, as for example a greater adhesion of platelets, have some role in it. However, in several clinical studies it has been demonstrated that the presence of platelets in the cluster of CD63-positive basophils is minor.^8^, ^9^, ^10^

**Fig. 1:**
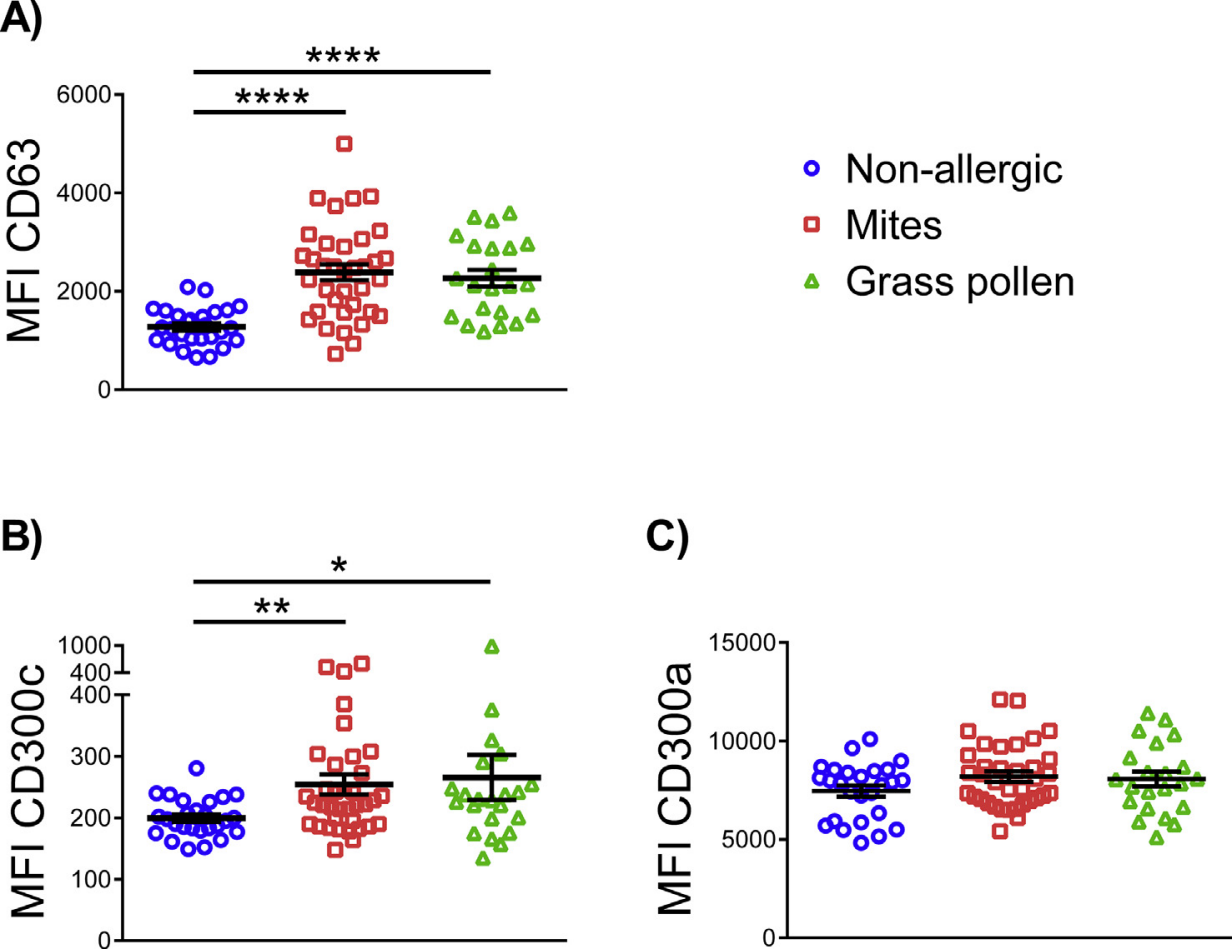
Basophils from allergic individuals exhibit greater expression of CD63 and CD300c. Dot graph showing basal median fluorescence intensity (MFI) of CD63 (A), CD300c (B) and CD300a (C) on basophils from non-allergic (blue), allergic to dust mites (red) and allergic to grass pollen (green) individuals. Each dot represents a donor, means ± SEMs are shown. *P < .05, **P < .01, ****P < .0001

Next, we analyzed cell surface expression levels of the inhibitory and activating CD300a and CD300c receptors, respectively. We observed that surface CD300c expression levels are significantly higher in basophils from individuals with an IgE-dependent allergy (dust mites and grass pollen) than in those from non-allergic individuals (Fig. 1B). This increased expression of CD300c could diminish basophils FcεRI-mediated activation threshold.^5^ It has been previously described that the basal expression of the CD300a inhibitory receptor is lower in basophils from patients with birch pollen allergy than in basophils from healthy control subjects.^11^ However, we have not seen significant differences between healthy and allergic individuals related to CD300a expression (Fig. 1C), which is in agreement with previous results in cow's milk allergic children.^5^

We performed correlation analysis between the intensity of CD300c and CD63 expression, but we did not find any significant result (data not shown). As it has been mentioned before, an association between the severity of the hypersensitivity symptoms and the levels of CD300c expression on basophils in cow's milk allergic children has been described.^5^ However, in this study the vast majority of the recruited patients (88%) were classified to have mild symptomatology (Table 1), and therefore it was not adequate to perform this analysis.

Unlike mite allergy, grass pollen allergy is a seasonal affection, and the exposure of patients to the allergen varies throughout the year. Although the great majority of samples have been collected during the grass season, we have two samples of the same individual collected at different times of the year, one in grass season and the other not. We observed that the expression of CD300c is higher during the grass pollen season than out of the season (MFI 492 vs 376, respectively). This may suggest that the exposure of allergic patients to higher amounts of allergen could induce an up-regulation of basophil activating receptors such as CD300c. The specific mechanism regulating CD300c expression in these allergic patients is still unknown and deserves further studies.

To date, the only tested stimulus able to upregulate CD300c expression is IL-3.^5^ This is a very important cytokine for the development, maturation, and survival of basophils.^12^ This cytokine is known to markedly increase the activation and release of mediators from basophils in IgE-dependent responses,^12^ and the autocrine priming with IL-3 has been described as an important mechanism behind the hyper-reactive nature of basophils in the allergic disease.^13^ We analyzed IL-3 in plasma from allergic subjects, but the levels of this cytokine were mostly undetectable. It is possible that the mild symptoms exhibited by the majority of patients may be related to the observed results. Based on our data, we propose that baseline expression levels of CD300c, together with CD63 expression, on human basophils could be helpful for the diagnosis of IgE-dependent allergies. Furthermore, considering that CD300c is capable of modulating the threshold of IgE-mediated activation in human basophils,^5^ we believe that, as previously demonstrated,^5^ an increased expression of CD300c decreases the IgE-dependent activation threshold of basophils in allergic individuals.

## Conflict of interests

The authors declare that the research was conducted in the absence of any commercial or financial relationships that could be construed as a potential conflict of interest.

## Consent for publication and ethics approval

Blood samples from healthy donors and allergic patients were collected through the Basque Biobank (http://www.biobancovasco.org). The Basque Biobank complies with the quality management, traceability, and biosecurity set out in the Spanish Law 14/2007 of Biomedical Research and the Royal Decree 1716/2011. All subjects provided written and signed informed consent in accordance with the Declaration of Helsinki. The protocol was approved by the Basque Ethics Committee for Clinical Research (PI2015182; 15–56; version 3; March 23, 2017).

## Figures and Tables

**Fig. S1:**
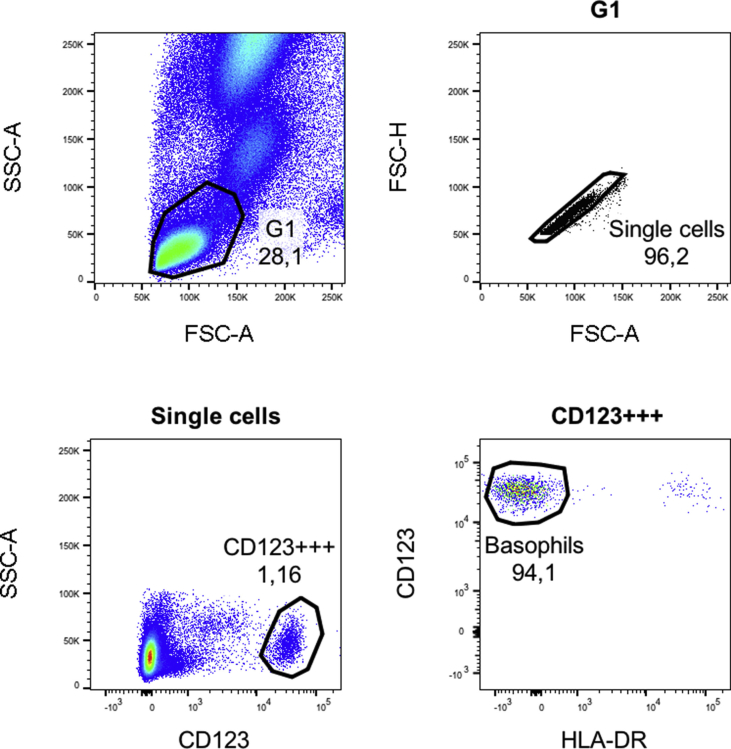


**Fig. S2:**