# Supplementary material for: Increased expression levels of CD300c on basophils from allergic individuals
Source: World Allergy Organ J. 2019 Sep 30;12(9):100060. doi: 10.1016/j.waojou.2019.100060 (PMC6796774; doi:10.1016/j.waojou.2019.100060)
Supplement: Multimedia component 1 [file mmc1.doc]

# SUPPLEMENTAL MATERIAL

**Methods**

**Samples and methods**

Blood samples from healthy donors and allergic patients were collected through the Basque Biobank (http://www.biobancovasco.org). All the patients included in the study presented IgE-mediated allergy symptoms, positive skin prick test responses, and *in vitro* specific IgE to *Dermatophagoides pteronyssinus* and *Phleum pratense* in the 6 months before blood extraction (Table I). Skin prick tests were performed with commercial extracts of *Dermatophagoides pteronyssinus* and *Phleum pratense* (Roxall Laboratory, Bilbao, Spain) following the recommendations of the European Academy of Allergy and Clinical Immunology [14]. The skin tests were regarded as positive if the wheal diameter was at least 3 mm. A positive control of histamine 10 mg/mL and a negative control of saline 0.9% were included. Specific IgE to *Dermatophagoides pteronyssinus* and *Phleum pratense* was measured with the ImmunoCAP FEIA system (Thermo Fisher Scientific, Uppsala, Sweden) according to the manufacturer’s instructions. Values higher than 0.35 kU/L were considered positive. The severity of the symptomatology was classified based on the GINA (Global Initiative for Asthma) guide for asthma symptoms and on the ARIA (Allergic Rhinitis and its Impact on Asthma) guide for rhinitis symptoms. The Basque Biobank complies with the quality management, traceability, and biosecurity set out in the Spanish Law 14/2007 of Biomedical Research and the Royal Decree 1716/2011. All subjects provided written and signed informed consent in accordance with the Declaration of Helsinki. The protocol was approved by the Basque Ethics Committee for Clinical Research (PI2015182; 15-56; version 3; March 23, 2017).

**Antibodies and reagents**

The following anti-human monoclonal antibodies (mAbs) were used for flow cytometric analysis: FITC anti-CD63 (clone H5C6), BV412 anti-CD123 (clone 9F5) and PerCP-Cy5.5 anti-HLA-DR (clone G46-6) from BD Biosciences; PE anti-CD203c (clone 97A6) and PE anti-CD300a (clone E59.126) from Beckman-Coulter; eFluor660 anti-CD300c (clone TX45) and PE-Cy7 anti-FcεRI (clone AER-37) from eBioscience.

**Flow cytometry analyses**

Whole blood samples from healthy donors and allergic patients were collected in sodium citrate containing tubes and were used to determine the expression of CD300c and other surface markers, such a CD63, on basophils. Briefly, 100 µl of whole blood was stained with the respective fluorochrome conjugated mAbs for 20 minutes at room temperature. Next, red blood cells were lysed using 1X BD FACS Lysing buffer for 15 min at room temperature. Then, cells were washed with PBS to remove unbound mAbs and further acquired in a FACSCanto II Flow cytometer (BD Biosciences). As a negative control, we used fluorescence minus one (FMO), which contains all the fluorochrome conjugated mAbs with the exception of the marker of interest. FMO control is used to identify and gate cells in the context of data spread due to the multiple fluorochromes in a given panel. Flow cytometry data were analyzed by using FlowJo software, version 10.0.7 (TreeStar).

**Enzyme-Linked Immunosorbent Assay (ELISA)**

The Invitrogen Human IL-3 ELISA kit (KHC0031) was used to measure soluble IL-3 levels in the plasma samples from non-allergic and allergic individuals. ELISA experiments were performed following the manufacturers’ protocol.

**Statistical Analysis and graphical representation**

GraphPad Prism software (version 8.0.1) was used for graphical representation and statistical analysis. Data were represented in dot plot graphs showing the means ± standard error of the mean (SEM). For comparison between healthy and allergic individuals, first, we removed from the analysis the outlier identified with the ROUT (Robust regression and Outlier removal) method, and then the non-parametric, unpaired Mann-Whitney rank test was used. *p<0.05, ** p<0.01, ***p<0.001, ****p<0.0001.

**Supplemental References**

[14] Dreborg S, Frew A. Position Paper: Allergen standardization and skin tests. Allergy 1993;48:49–54. doi:10.1111/j.1398-9995.1993.tb04756.x.

**Figure legends**

**FIGURE S1:** Basophil´s gating strategy based on the expression of the surface receptor CD123 and the absence of HLA-DR.

**FIGURE S2:** Basal expression of CD63 on basophils from non-allergic (blue histogram), allergic to dust mites (red histogram) and allergic to grass pollen (green histogram) individuals. A representative example from data shown in Figure 1A is shown.
